# Effects of Lutein/Zeaxanthin Supplementation on the Cognitive Function of Community Dwelling Older Adults: A Randomized, Double-Masked, Placebo-Controlled Trial

**DOI:** 10.3389/fnagi.2017.00254

**Published:** 2017-08-03

**Authors:** Billy R. Hammond, L. Stephen Miller, Medina O. Bello, Cutter A. Lindbergh, Catherine Mewborn, Lisa M. Renzi-Hammond

**Affiliations:** ^1^Department of Psychology, University of Georgia Athens, GA, United States; ^2^Bio-Imaging Research Center, Paul D. Coverdell Center for Biomedical and Health Sciences, University of Georgia Athens, GA, United States

**Keywords:** Xanthophylls, cognition, older adults, attention, cognitive flexibility

## Abstract

**Background:** High levels of xanthophyll carotenoids lutein (L) and zeaxanthin (Z) in the central nervous system have been previously correlated with improved cognitive function in community-dwelling older adults. In this study, we tested the effects of supplementing L and Z on older men and women with a range of baseline cognitive abilities.

**Objective:** The purpose of this study was to determine whether or not supplementation with L+Z could improve cognitive function in community-dwelling, older adults.

**Design:** Double-masked, randomized, placebo-controlled trial. A total of 62 older adults were randomized into groups receiving either 12 mg L+Z or a visually identical placebo. Data from 51 participants (*M* = 73.7 years) were available for analysis. Retinal L+Z levels (macular pigment optical density, MPOD) were measured psychophysically using heterochromatic flicker photometry as a biomarker of cortical L+Z levels. Cognitive function was measured using the CNS Vital Signs computerized test platform.

**Results:** Participants receiving the active L+Z supplement had statistically significant increases in MPOD (*p* < 0.03) and improvements in complex attention (*p* < 0.02) and cognitive flexibility domains (*p* < 0.04), relative to participants taking the placebo. A trend was also seen for the executive function domain (*p* = 0.073). In male participants only, supplementation yielded improved composite memory (*p* = 0.04).

**Conclusions:** Supplementation with L+Z improved cognitive function in community-dwelling, older men and women.

## Introduction

It has long been understood that cognitive function, along with its manifestation as behavior and subjective experience, is a product of the activity of the brain. Hebb (1949), for instance, argued that it was the interplay between networks of neurons that gave rise to mental activity. Further, deleterious change within neural cell assemblies drives many of the decrements often observed with aging (e.g., Berlingeri et al., 2013). For example, age-related change in neural cells^1^ means that older individuals must activate larger regions of these networks in order to accomplish the same task as a younger person. This additional recruitment results in slowing and the common finding that dynamic aspects of cognition are more impacted by age than more static functions (Salthouse, 1996).

If, however, these neural assemblies are the physical basis of cognition and its age-related change, then if follows that the physical factors involved in forming and maintaining those physical structures would, inevitably, influence the end-product itself. Such factors are often systemic. For example, interferon-Y, a component of the immune system, has recently been shown to regulate neural connectivity and social behavior in mice (Filiano et al., 2016). Many neurotransmitters are synthesized within the gut, and the physical structure of the brain itself is dependent on dietary and immune factors originating in this distal tissue (Cryan and Dinan, 2012). In an environment of high fat (some 60% by volume) and oxygen (25% respiratory intake), the brain must concentrate high levels of antioxidants (both dietary and endogenous, like superoxide dismutase) to prevent peroxidation (Chakrabarti et al., 2011). If antioxidants are missing in the diet, then higher levels of oxidative stress exist within the brain (Rao and Balachandran, 2002). Over time, peroxidation of brain lipids likely results in losses such as the decrease in the quantity and integrity of white matter often seen with aging (Bennett and Madden, 2014), likely due to alterations in the lipid-rich axonal myelin.

Lutein, a dietary antioxidant, could help maintain brain structure by lowering chronic oxidative stress (Erdman et al., 2015). The brain is also susceptible to damage due to chronic inflammation and L and Z are known to be potent anti-inflammatories (Kijlstra et al., 2012). Such mechanisms, however, are largely prophylactic. It is reasonable to question then whether preventive measures are effective later in life. Is there value, for instance, in increasing dietary intake of food components thought to prevent loss after someone has likely already suffered many decades of loss?

We do know that older and diseased brains (and retinas) are under higher oxidative and inflammatory stress. Late stage intervention could possible lower such stressors helping to retard the cascade that ultimately accelerates the degenerative process (Hammond et al., 1998; Joseph et al., 2005). We also know that older brains are still capable of some neurogenesis (especially within the hippocampus; Kempermann et al., 2002). If supplemental L, through diet or purified supplements, could both decrease age-related inflammatory and oxidative stress while simultaneously stimulating regenerative processes, supplementing L certainly could be a useful strategy. There is some, limited, data that are consistent with this possibility.

For instance, preliminary data suggests that, in younger individuals, supplementing L and Z increases systemic levels of brain-derived neural growth factor (Stringham et al., 2016) when compared to placebo. A number of clinical trials have shown, mostly in the young, that L and Z supplementation increases visual processing speed and reaction times (Bovier et al., 2014; Bovier and Hammond, 2015). Although there is no direct data on mechanism, it has been speculated (e.g., the neural efficiency hypothesis for L and Z) that this influence on processing speed is due to direct effects on brain connectivity (Renzi and Hammond, 2010), perhaps by enhancing gap junctions between neurons.

Whatever the mechanism, we do have empirical data on participants across the lifespan showing that L and Z supplementation has direct effects on improving cognition compared to placebos. Johnson et al., for instance, showed that L and Z, combined with DHA, improved verbal fluency, rate of learning and memory (Johnson et al., 2008). Later, this basic finding was repeated using avocados, rich in lutein and omega-fatty acids (Johnson et al., 2015). In the current study, we extend these basic findings, also using a double-blinded placebo controlled design, to test both older men and women using only L and Z (separating it from the much larger literature on omega fatty acids and cognition).

## Materials and methods

### Subjects

A total of 80 community dwelling older adults from the Athens-Clarke County, Georgia population were screened for enrollment between August 2012 and August 2014, with follow-up lasting through October 2015. This sample was part of a larger trial on xanthophyll supplementation and cognitive function. Inclusion criteria included good overall health; no xanthophyll supplementation within the 6-month period prior to study enrollment, with the exception of multivitamins that contained less than 1 mg L+Z/day; best corrected visual acuity of 20:40 or better (Snellen notation); no previous history of stroke, dementia, Parkinson's disease, or any other neurological condition known to impair cognitive function, with the exception of affective disorders such as anxiety or depression; absence of gastric conditions known to impair absorption of nutritional supplements, such as gastric bypass or gastric ulcer. Inclusion criteria were verified as follows: all participants were given a medical examination by a qualified physician at the University of Georgia Health Center; self-reported health information was obtained; and all participants participated in a structured clinical interview, administered by qualified neuropsychological staff (see below).

### Randomization process and intervention

Of the 80 participants that were screened for enrollment, a total of 62 participants met inclusion criteria and were randomized into one of two groups: the active supplement group, or the placebo group. Simple randomization was conducted by the clinical coordinator, who had no data collection responsibilities. A set of numerical codes was generated that corresponded with either the active supplement or the placebo. The codes were placed in an opaque envelope, and a unique code was drawn for each participant. Of the 62 participants who were randomized, 20 participants were randomized into the placebo group, and 42 participants were randomized into the active supplement group. In the placebo group, two were lost to follow-up (completed baseline but failed to attend subsequent testing sessions) and three were withdrawn due to non-compliance. In the intervention group, four were lost to follow-up and one was withdrawn.

The active supplement contained 10 mg L and 2 mg Z. The placebo was visually identical to the active supplement. Supplements and placebos (provided by DSM Nutritional Products Ltd., Kaiseraugst, Switzerland) were contained in identical opaque, sealed bottles with labels that were visually identical, with the exception of the randomization code on the label, and contained instructions for one tablet to be taken from the bottle, daily, with a meal. Compliance to the intervention was monitored by bi-monthly telephone calls and pill counts from bottles returned by the participants during study visits.

### Baseline characteristics of the analyzable sample

Of the 62 participants who were randomized, a total of two participants from the placebo group and four participants from the active supplement group were lost to follow-up over the year of intervention. Three participants from the placebo group and 1 participant from the active supplement group were withdrawn by study personnel for either failure to maintain inclusion criteria, or because of non-compliance with the study regimen, determined by self-reported failure to take study supplements on a minimum of four of the compliance telephone calls. Baseline characteristics of the analyzable sample (*N* = 51) are presented in Table 1. There were no significant differences between the placebo and active supplement groups in gender distribution, age (*p* > 0.48), or education level (*p* > 0.89).

**Table 1:** Baseline characteristics of the analyzable study sample.

**Study group**	**Age (years)**	**Gender**	**Years of education**	**Dietary intake of fruits and vegetables (servings/day)**	**Baseline macular pigment optical density (MPOD)**
All analyzable participants	73.74 ± 8.20	30 female; 21 male	16.34 ± 3.01	5.26 ± 1.48	0.49 ± 0.18
Active Supplement Group	72.51 ± 6.24	19 female; 17 male	16.37 ± 3.21	5.25 ± 1.40	0.51 ± 0.19
Placebo Group	70.93 ± 5.70	11 female; 4 male	16.25 ± 2.53	5.26 ± 1.56	0.42 ± 0.16

*All participants reported ethnic and racial information as “Non-Hispanic/White.” Values are presented as Mean, ± Standard Deviation*.

With respect to cognitive function, significant differences were detected at baseline between male (*M* = 88.19, *SD* = 8.79) and female (*M* = 96.60, *SD* = 7.61) participants in the composite memory domain (*p* < 0.05). No other significant differences were present between male and female participants at baseline.

### Ethics

The tenets of the Declaration of Helsinki were adhered to at all times during the course of this study. All participants issued written and verbal informed consent prior to study enrollment, and consent documents were administered by trained study personnel. The University of Georgia Institutional Review Board approved all study-related documents and procedures prior to study initiation, and all study personnel received training in ethical principles and procedures in human subject's research.

### Methods

#### Confirming enrollment criteria

In order to be enrolled in this study, participants went through a three-step process to confirm eligibility. Participants were initially recruited using newspaper advertising and print advertisements posted throughout the community. When participants contacted study personnel to express interest, a telephone screener was used to collect self-report data on past supplement use and a brief history of neurological and ocular disorders. If the participant passed the first eligibility screen, visits were scheduled with the University of Georgia Health Center for the medical examination, as well as the University of Georgia Neuropsychology and Memory Assessment Laboratory. If the study medical team confirmed eligibility, the participant progressed to the neuropsychological screen / clinical interview.

Participants were asked to bring a legally authorized representative to the interview, who could serve as a collateral source of information. In addition to baseline cognitive functional testing on the primary test battery (CNS Vital Signs; Morrisville, NC), participants and collaterals were given the Clinical Dementia Rating Scale (CDR) as part of the larger interview (Morris, 1993). Potential participants with CDR sum of boxes scores of 1.0 or higher were excluded from participation. Participants with sums of boxes equal to 0.5 (mild impairment, O'Bryant et al., 2008) were included in the study sample, in order to include participants with a wider range of baseline cognitive abilities.

#### Retinal L ± Z levels

Retinal L+Z levels, as macular pigment optical density (MPOD) were measured psychophysically using customized heterochromatic photometry (cHFP) (Wooten et al., 1999; Stringham et al., 2008). This procedure has been described previously (Vishwanathan et al., 2014) and was modified slightly for this study. First, rather than the five trials per condition that are typically used to measure macular pigment, nine trials were completed both centrally, at 30-min of eccentricity along the horizontal meridian of the temporal retina, and parafoveally, at 7° of eccentricity. These trials were completed using a test stimulus that consisted of a waveband peaking at 460 nm (strongly absorbed by MP) that alternated in counterphase with a reference waveband peaking at 570 nm. Data collection was also limited to three skilled experimenters.

cHFP is the gold standard for measuring MPOD (Hammond et al., 2005) and has been used previously in participants with poor ocular health (e.g., with cataract and age-related macular degeneration) (Ciulla et al., 2001; Stringham et al., 2008), children (McCorkle et al., 2015), and participants with mild cognitive impairment (Renzi et al., 2014). In order to further confirm reliability of cHFP in this sample, an additional five central trials and five parafoveal trials were conducted using a different test stimulus, which consisted of a waveband peaking at 490 nm that alternated in counterphase with the same 570 nm reference waveband. Past research using *ex vivo* absorption spectra (Snodderly et al., 1984) and *in vivo* psychophysical methods (Snodderly et al., 2004; Wooten and Hammond, 2005; Stringham et al., 2008) for MP suggests that at 490 nm, absorbance is reduced by approximately half. Consequently, MPOD using the 490 nm test stimulus should be approximately half of the value at 460 nm if participants were successfully able to understand and complete the psychophysical task.

#### Serum L ± Z levels

In addition to measuring retinal L+Z levels, which relate strongly to cortical L+Z levels in human subjects (Vishwanathan et al., 2016) and are used as a biomarker of cortical L+Z in this and other studies (e.g., Feeney et al., 2013; Renzi et al., 2014; Vishwanathan et al., 2014), L and Z were also measured in serum via high-performance liquid chromatography. The methods used to acquire and analyze the serum in this study have been prevented previously (Lindbergh et al., 2017).

#### Cognitive function

Cognitive function was measured using a computerized test battery (CNS Vital Signs; Morrisville, NC) at four different time points: baseline, and after 4-, 8-, and 12-months of taking the study intervention. Participants were tested in a low distraction environment, in full ambient room lighting, in the presence of a trained research assistant who could answer questions about the test procedures should they arise. Each participant completed a practice session prior to each individual test.

Raw scores from individual functional tests taken during the test session were used to compute performance on the following larger cognitive domains: Verbal Memory (VeM), Visual Memory (ViM), Reasoning Ability (R), Executive Function (EF), Psychomotor Speed (PmS), Cognitive Flexibility (CF), and the Neurocognitive Index (NCI). For example, in order to gauge performance in CF, errors on the shifting attention test and commission errors on the Stroop task were subtracted from correct responses on the shifting attention test. For a complete list of cognitive tests administered during the battery, as well as functional domains computed and analyzed, see Table 2.

**Table 2:** Individual tests administered during the computerized cognitive functional test battery, and computed domain scores analyzed for older adult participants.

**Domain**	**Description**	**Tests used to compute the domain**	**Computation**
Verbal Memory (VeM)	Ability to remember words presented in a list vs. distractor words, immediately after list presentation and after a 30-minute delay.	Verbal Memory Test	Correct hits for presented words + correct passes on distractors for tests immediately after presentation and after a 30-min delay.
Visual Memory (ViM)	Ability to remember arbitrary visual shapes and symbols vs. distractor shapes and symbols immediately after presentation and after a 30-minute delay.	Visual Memory Test	Correct hits for presented shapes and symbols + correct passes on distractors for tests immediately after presentation and after a 30-min delay.
Reasoning ®	Ability to perceive and understand the meaning of abstract concepts and recognize the relationships between abstract concepts.	Non-Verbal Reasoning Test (NVRT)	Correct responses on the NVRT – commission errors on the NVRT.
Executive Function (EF)	Ability to recognize and act upon sets with randomly shifting rues in the presence of other simultaneously occurring tasks and pieces of information.	Shifting Attention Test (SAT)	Correct responses on the SAT – errors on the SAT.
Psychomotor Speed (PmS)	Ability to rapidly preform motor tasks in absence of sensory stimuli.	Finger Tapping Test (FTT) Symbol-Digit Coding Test (SDC)	Average number of taps on the FTT with the right hand + average number of taps with the left hand + number of correct responses on the SDC
Complex Attention (CA)	Ability to maintain sustained attention or vigilance in the face of changing response rules	Stroop Test (ST) SAT Continuous Performance Task (CPT)	Commission errors on the ST + Errors on the SAT + Commission and omission errors on the CPT
Cognitive Flexibility (CF)	Ability to inhibit irrelevant information and disinhibit previously “incorrect” response patterns.	SAT ST	Correct responses on the SAT – errors on the SAT – Commission errors on the ST
Neurocognitive Index (NCI)	Global cognitive functioning, takes into account all other functional domains	N/A	Average of domain scores from: ViM and VeM, PmS, Reaction Time across domains, CA, CF

#### Statistical analyses

Statistical analyses were performed using SPSS version 23 (IBM), with α = 0.05. Tests of cognitive function were one-tailed, as *a priori* hypotheses were directional in nature (i.e., increasing MPOD by supplementation will improve cognitive function). Prior to enrolling subjects, a power analysis was conducted to determine what sample size would yield 1−β = 0.80 for a difference of 0.10 log units of MPOD. The current sample size yielded statistical power of 85.7%. A statistically significant increase in MPOD in the supplementation group was analyzed as the primary outcome variable in this study, and improvements in cognitive function in the supplemented group relative to the placebo group were analyzed as secondary variables.

## Results

### Retinal L ± Z levels

At baseline, MPOD for the entire older adult cohort (0.49) was comparable to other published data on a different sample with approximately the same average age, recruited from the same geographic region (0.47) (Renzi et al., 2014), but was higher than MPOD reported in similarly aged cohorts from other parts of the U.S. (0.34, 0.36; Moeller et al., 2009; Vishwanathan et al., 2014) and world (0.20; Feeney et al., 2013). MPOD at baseline was numerically but not statistically higher in the group that received the active supplement (0.51 ± 0.19) than the group that received the placebo (0.42 ± 0.16). MPOD increased significantly between the baseline and the 12-month time points (*M* = 0.58, *SD* = 0.23; *p* < 0.03) in the group that received the active supplement. The placebo group did not change significantly over the course of the year (see Table 3).

**Table 3:** Serum levels of L, Z, and L+Z at baseline and over the course of the study intervention, stratified by intervention group.

		**Baseline**	**4-months**	**8-months**	**12-months**
Lutein (ng/μL)	Active	0.15 ± 0.08	0.66 ± 0.34^**^^†^	0.55 ± 0.27^**^^†^	0.59 ± 0.23^**^^†^
	Placebo	0.15 ± 0.06	0.25 ± 0.17	0.17 ± 0.09	0.14 ± 0.07
Zeaxanthin (ng/μL)	Active	0.03 ± 0.02	0.15 ± 0.09^**^^†^	0.12 ± 0.05^**^^†^	0.13 ± 0.06^**^^†^
	Placebo	0.03 ± 0.01	0.04 ± 0.03	0.03 ± 0.01	0.03 ± 0.01
Lutein + Zeaxanthin (ng/μL)	Active	0.18 ± 0.11	0.81 ± 0.39^**^^†^	0.66 ± 0.32^**^^†^	0.72 ± 0.28^**^^†^
	Placebo	0.18 ± 0.07	0.29 ± 0.20	0.21 ± 0.10	0.17 ± 0.08
Macular Pigment (optical density)	Active	0.52 ± 0.19	0.51 ± 0.18	0.58 ± 0.22^*^^†^	0.59 ± 0.22^†^
	Placebo	0.42 ± 0.16	0.39 ± 0.21	0.38 ± 0.17	0.47 ± 0.20

*Data are presented as M ± SD*.

* *Denotes significant difference between active and placebo group (p < 0.05)*.

** *Denotes a significant difference between active and placebo group (p < 0.01)*.

† *Denotes a significant change from baseline (p < 0.05)*.

When MPOD at 460 nm was compared against MPOD at 490 nm to confirm validity at the 12-month timepoint, total MPOD for the study sample, regardless of group membership, was 0.53 at 460 nm. When measured at 490 nm, MPOD was 0.29, which is approximately half the 460 nm value, suggesting that participants were able to reliably perform the cHFP task.

### Serum L ± Z levels

**Figure 1:**
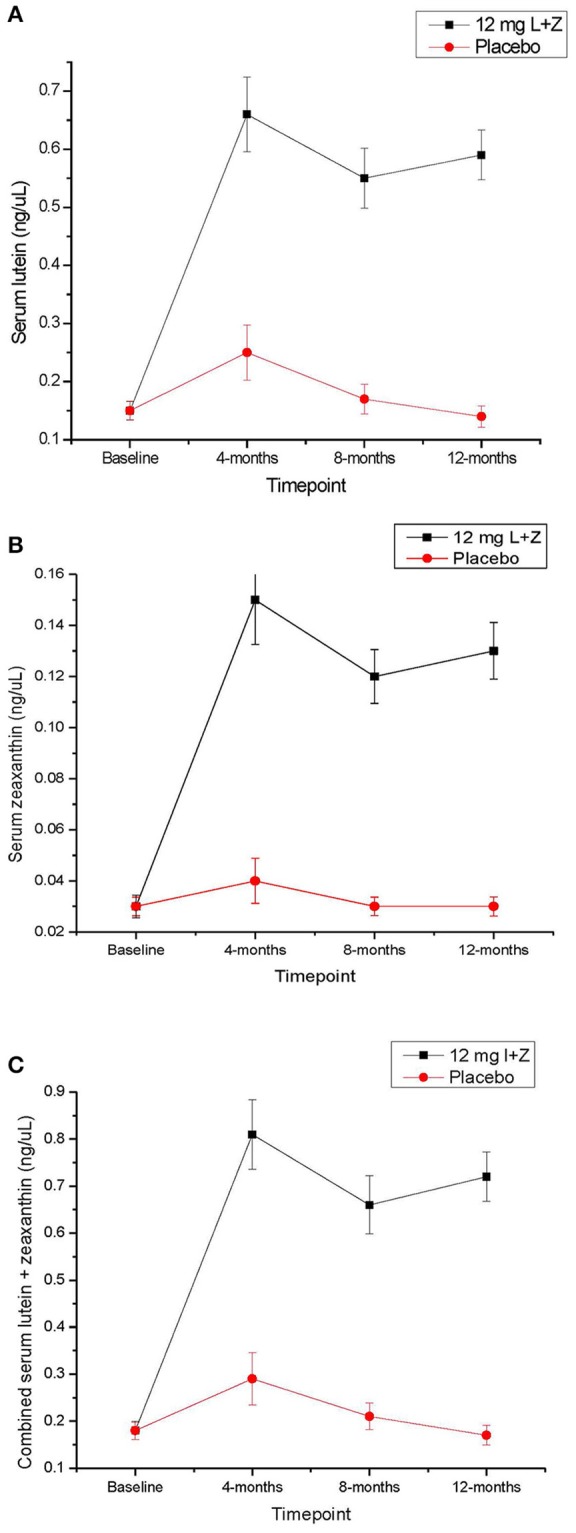
**(A)** Serum lutein across the 1-year intervention time period in participants taking the active study supplement, vs. placebo. Error bars represent the standard error of the mean. **(B)** Serum zeaxanthin across the 1-year intervention time period in participants taking the active study supplement, vs. placebo. Error bars represent the standard error of the mean. **(C)** Serum lutein + zeaxanthin levels across the 1-year intervention time period in participants taking the active study supplement, vs. placebo. Error bars represent the standard error of the mean.

At baseline, serum L, Z, and L+Z levels were not significantly different between participants in the active supplement and placebo groups. Beginning at the 4-month time point and continuing throughout the rest of the intervention, serum L, Z, and L+Z were significantly higher in the group that received the active supplement than the placebo group (*p* < 0.01 for L, Z, and L+Z at all-time points; see Table 3, Figure 1). Participants on the placebo supplement did not show any significant changes in serum L, Z, or L+Z levels during the year-long intervention.

### Cognitive function

At baseline, participants in the active supplement group were not significantly different from participants who were randomized into the placebo group on any of the cognitive domain scores analyzed or on global cognitive health, as measured by the CDR (see Table 4). At baseline, trends for relationships between MPOD and the memory, executive function and cognitive flexibility domains were present in the sample as a whole, but not statistically significant (*p* > 0.05). Given the relatively low sample size present in this study, the lack of statistical significance is not surprising.

**Table 4:** Cognitive domain scores at baseline for the entire sample, and for the sample stratified by supplement status.

	**NCI**	**VeM**	**ViM**	**R**	**EF**	**PmS**	**CA**	**CF**	**CDR = 0.5**
Whole Sample	101.10 ± 10.16	50.76 ± 5.46	42.98 ± 9.66	2.82 ± 3.88	33.41 ± 17.33	141.86 ± 19.84	12.27 ± 10.15	31.83 ± 18.23	7.69% of sample
Active Supplement Group	100.12 ± 10.45	49.91 ± 5.66	41.03 ± 6.68	2.97 ± 3.95	32.33 ± 18.33	140.24 ± 20.11	13.12 ± 11.12	30.32 ± 19.09	8.8% of sample
Placebo Group	103.33 ± 9.44	52.67 ± 5.29	43.87 ± 5.18	2.47 ± 3.83	35.87 ± 15.90	145.53 ± 19.37	10.21 ± 7.21	35.50 ± 16.01	6.67% of sample

*NCI, Neurocognitive Index; VeM, Verbal Memory; ViM, Visual Memory; R, Reasoning Ability; EF, Executive Function; PmS, Psychomotor Speed; CA, Complex Attention; CF, Cognitive Flexibility; CDR, Clinical Dementia Rating Scale*.

*Data are presented as M ± SD*.

When correlations between MPOD and cognitive function were analyzed at the 12-month time point, MPOD was significantly related to performance in the reasoning domain (*r* = 0.45, *p* = 0.04), and a trend was seen for errors of attention in the complex attention domain (*r* = −0.18, *p* = 0.08). A trend for relation between MPOD and verbal memory (*r* = 0.31, *p* = 0.07) was also present for those participants whose MPOD improved from the baseline to the 12-month time point, regardless of whether or not they received the supplement. Within the supplementation group, participants with the greatest changes between the baseline and 12-month time points in cognitive function in the reasoning (*r* = 0.34, *p* = 0.02) and complex attention (*r* = −0.31, *p* = 0.04, expressed as errors in complex attention) domains also tended to have the highest MPOD at the 12-month time point. Trends for the relationship between magnitude of cognitive change and 12-month MPOD were also seen for the visual memory (*r* = 0.24, *p* = 0.09) and cognitive flexibility (*r* = 0.20, *p* = 0.10) domains.

**Figure 2:**
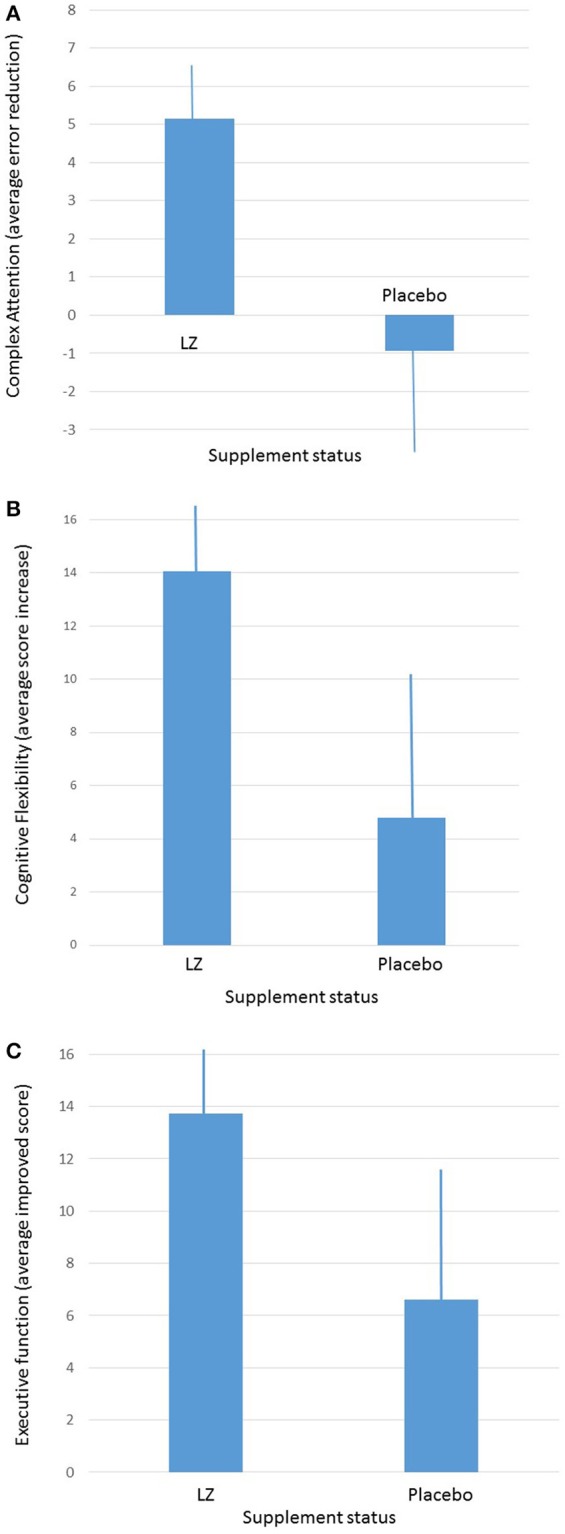
**(A)** Average improvements in complex attention between participants taking the active supplement and participants taking the placebo after 1-year of intervention. **(B)** Average improvements in cognitive flexibility between participants taking the active supplement and participants taking the placebo after 1-year of intervention (average and ± SEM). **(C)** Average improvements in executive function between participants taking the active supplement and participants taking the placebo after 1-year of intervention (average and ± SEM).

Given the fact that participants were tested four times throughout the course of the study, and given the fact that participants were given practice sessions prior to each active test session, practice effects were anticipated. In order to determine whether or not change was reliable and meaningful, the Reliable Change Index (RCI) was computed for each cognitive index and group, with a standard criterion of 1.96. Participants who took the active supplement had significantly improved performance in complex attention (*p* < 0.02; RCI = 3.71 for the active group and 0.34 for the placebo group) and cognitive flexibility (*p* < 0.04; RCI = 6.31 for the active group and 0.84 for the placebo group) domains, relative to participants taking the placebo. A trend was also present (*p* = 0.07; RCI = 5.64 for the active group and 1.27 for the placebo group) for the executive function domain (see Figure 2), by the end of the 12-month period. When male and female participants were analyzed separately, male participants who received the active supplement improved significantly in the composite memory domain (*p* = 0.04).

## Discussion

This study was designed as a year-long intervention with the dietary carotenoids L and Z. These plant pigments, long known for their effects on systemic and ocular health, have been identified in brain (Craft and Dorey, 2004; Vishwanathan et al., 2016). Accumulating evidence has shown that LZ may influence various aspects of brain function ranging from visual-motor to executive functions (e.g., Johnson et al., 2008; Feeney et al., 2013; Bovier et al., 2014; Renzi et al., 2014; Vishwanathan et al., 2014). L and Z, being lipid-soluble, easily pass the blood-brain and blood-retinal barriers and tend to deposit within central nervous system tissues with high specificity (e.g., in the retina, they concentrate toward the central macular region). Past study has shown that brain concentrations of L associate with higher cognitive test scores in the elderly (Johnson et al., 2013) prompting the possibility that increasing intake could lead to benefit. Johnson et al., first in 2008 using purified supplements (Land DHA), and then again in 2011 using whole food (avocados), confirmed that such interventions could lead to improved cognitive function in older subjects (Johnson et al., 2008, 2015). In the current study, we also found that, when compared to placebo, supplementing 10 mg of L and 2 mg of Z for one year led to statistically significant increases in complex attention and cognitive flexibility (with numerical increases, but not exceeding statistical criteria for significance, *p* < 0.07, in executive function) in a sample of older adults.

In many respects, it is quite surprising that simple dietary change can lead to any improvements when considering such a homogeneous (Caucasian, upper middle-class), well-nourished and educated, group such as was sampled in our study. As with any dietary experiment, there were no true placebos, as the term is typically used in pharmaceutical studies: subjects have been exposed to the “intervention” all of their life (meaning that L is present in many normally consumed foods). Even during the intervention year, the control group maintained their normal diet which contains L and Z. Hence, any effect of L and Z on the treatment group would therefore have to be simply additive. Using this framing, the research question becomes: does adding LZ in supplement form to relatively well-nourished subjects with normal LZ intake improve cognition in a sample that was already well-educated? Education tends to attenuate any relation between diet and cognition (Akbaraly et al., 2009), mostly because more educated people tend to be well-fed (hence, our relatively high baseline MP levels) and dietary effects tend to be driven by deficiency (enhancement from normal is always much harder to achieve). The optimal sample for these studies are subjects who are less well fed, more diverse, less educated, etc. Similarly, an optimal intervention would likely include whole foods as opposed to supplements. Further, our study results, like many, are limited by convenience sampling/interventions which turns out to be, of course, of limited convenience when it comes to interpreting the actual real world effects of changing diet. Given these kind of limitations, the fact that L and Z did yield some benefit, especially when coupled with the results from other labs showing similar effects, suggests that L and Z do, in fact, have a positive effect on higher level functions of the brain.

## Author contributions

Authors LR, BH, and LM contributed to research study design; LR, BH, LM, CL, and CM collected study data; MB served as clinical coordinator; LR, BH, and LM contributed to data analysis; LR and BH drafted the initial manuscript; LH, BH, and LM primarily edited the manuscript; LR, BH, LM, CL, CM, and MB assume responsibility for the final content of the manuscript.

## Registry information

ClinicalTrials.gov number, NCT02023645.

## Disclosure

During a portion of data collection, author LR was employed by Abbott Nutrition. LR is now solely employed by the University of Georgia.

### Conflict of interest statement

Authors LR and BH have received honoraria from Abbott Nutrition for presentation of research findings. The other authors declare that the research was conducted in the absence of any commercial or financial relationships that could be construed as a potential conflict of interest.

## Footnotes

^1^This appears to be less the result of neuronal death per se and more due to actual changes in morphology; for example, Hof and Morrison (2004) showed a 46% decrease in dendritic spine number and density when comparing younger and older adults.

## Abbreviations

L: Lutein
Z: zeaxanthin
MPOD: macular pigment optical density
MZ: meso-zeaxanthin
CNS: central nervous system
MP: macular pigment
HPLC: high-performance liquid chromatography
FFQ: food frequency questionnaire
RCI: reliable change index
VeM: verbal memory
ViM: visual memory
R: reasoning
EF: executive function
PmS: psychomotor speed
CA: complex attention
CF: cognitive flexibility
NVRT: non-verbal reasoning test
SAT: shifting attention test
FTT: finger tapping test
SDC: symbol-digit coding
ST: Stroop test
CPT: continuous performance task
CDR: Clinical Dementia Rating Scale
NCI: Neurocognitive index.
